# Supplementary material for: Secondary bone marrow graft loss after third-party virus-specific T cell infusion: Case report of a rare complication
Source: Nat Commun. 2024 Mar 29;15:2749. doi: 10.1038/s41467-024-47056-3 (PMC10980733; doi:10.1038/s41467-024-47056-3)
Supplement: Supplementary file 1 — Supplementary Information [file 41467_2024_47056_MOESM1_ESM.pdf]

Supplemental Material for:

Secondary bone marrow graft loss after third-party virus-specific T cell infusion: Case report of a rare complication

Michael D. Keller, [1, 2, 3.] Stefan A. Schattgen, [4] Shanmuganathan Chandrakasan, [5] E. Kaitlynn Allen, [4] Mariah A. Jensen-Wachspress, [1] Christopher A. Lazarski, [1] Muna Qayed, [5] Haili Lang, [1] Patrick J. Hanley, [1, 3, 6] Jay Tanna, [1, 6] Sung-Yun Pai, [7] Suhag Parikh, [5] Seth I. Berger, [8] Stephen Gottschalk, [4] Michael A. Pulsipher, [9] Paul G. Thomas, [4] Catherine M. Bollard* [1,3,6]

*Corresponding author: Catherine M. Bollard, MD
Email: cbollard@childrensnational.org

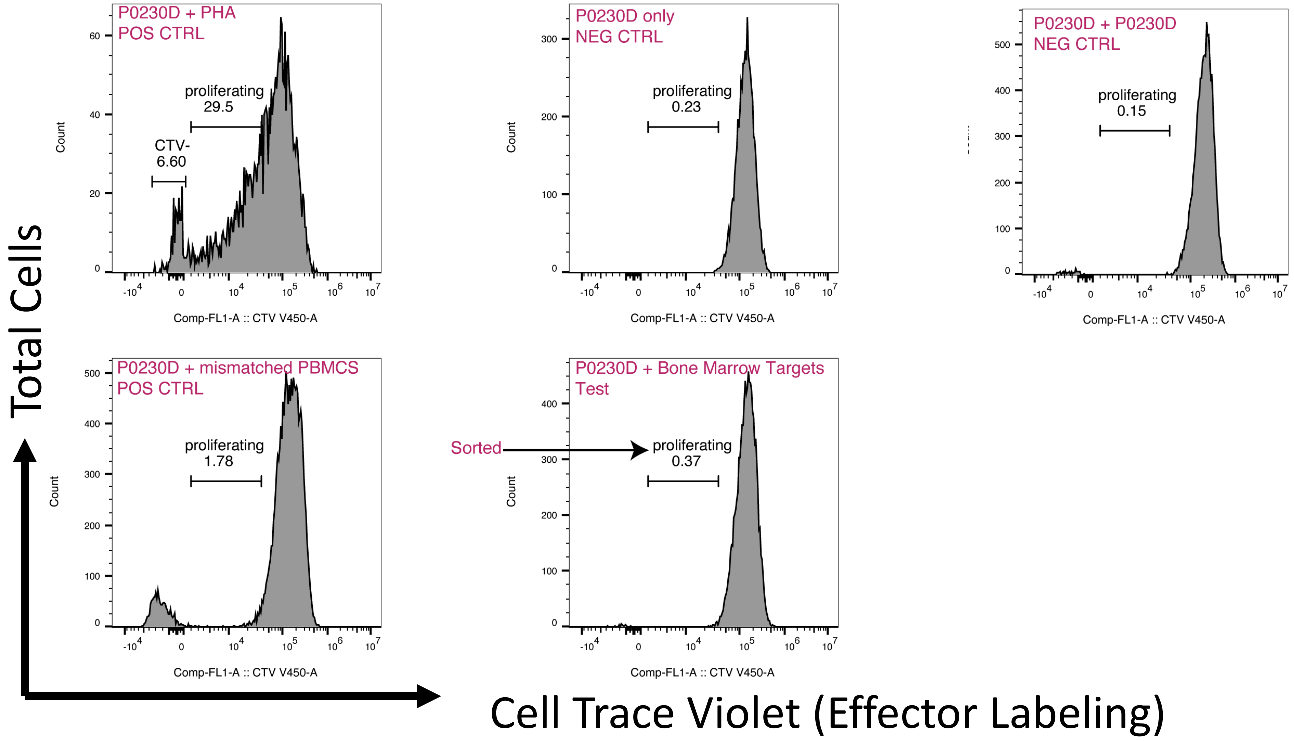

Supplemental Figure 1: Mixed lymphocyte culture of the infused VST product (P0230D) against irradiated target cells. Proliferation of VSTs by cell trace violent following 5 day co-culture with irradiated targets (HLA mismatched PHA blasts, autologous cells, or bone marrow product cells from the first BMT donor [paternal]). CTV = cell trace violet.

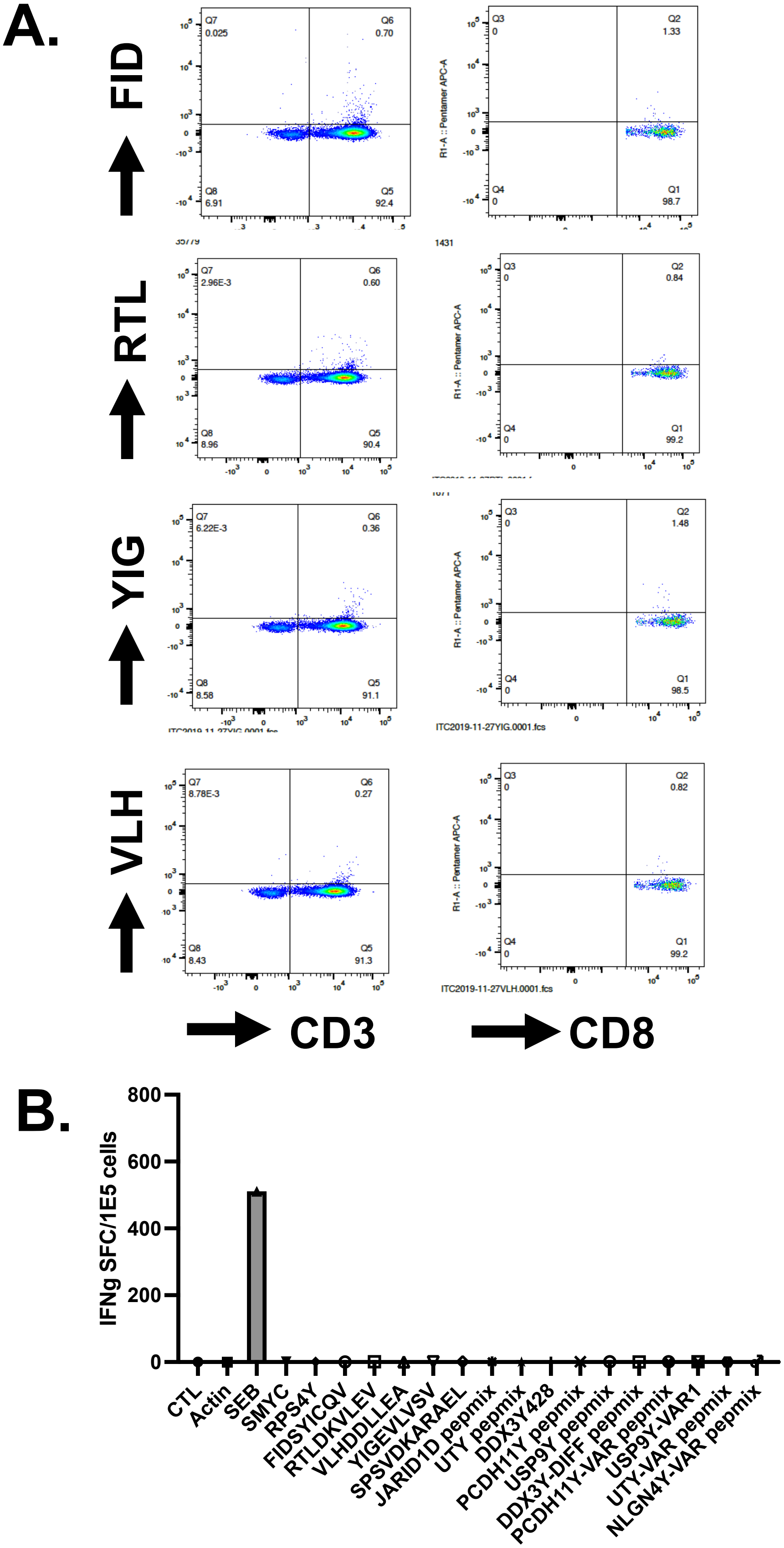

Supplemental Figure 2: Candidate alloantigen testing in the infused VST product (n=1). A. Pentamer staining for multiple previously described H-Y alloantigens in the VST product (FID: FIDSYICQV; RTL: RTLDKVLEV, VLH: VLHDDLLEA, YIG: YIGEVLVSV) B. Candidate alloantigen reactivity testing of the infused VST product by interferon-gamma ELISpot. SEB = staphylococcal enterotoxin B; SFC = spot forming colonies.

P0230D/CD4 T-cells, day 10 post-expansion

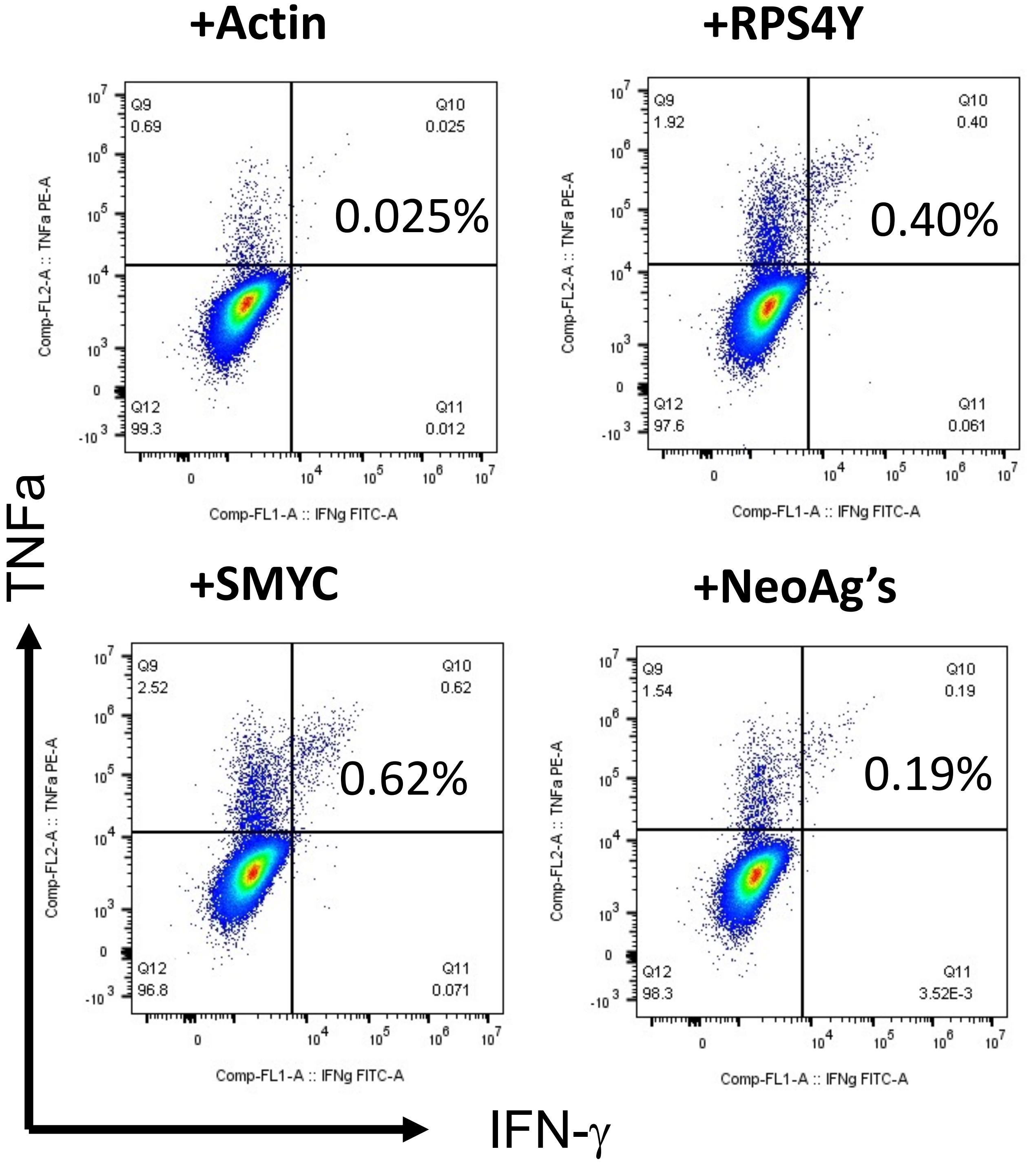

Supplemental Figure 3: T cell specificity testing for candidate alloantigens and neoantigens recognized by T cells from VST donor P0230D by intracellular flow cytometry at day 10 post-expansion. Neoantigen (NeoAg) peptide library was derived from the genome sequence of P0230D, as listed in Supplemental Table 7.

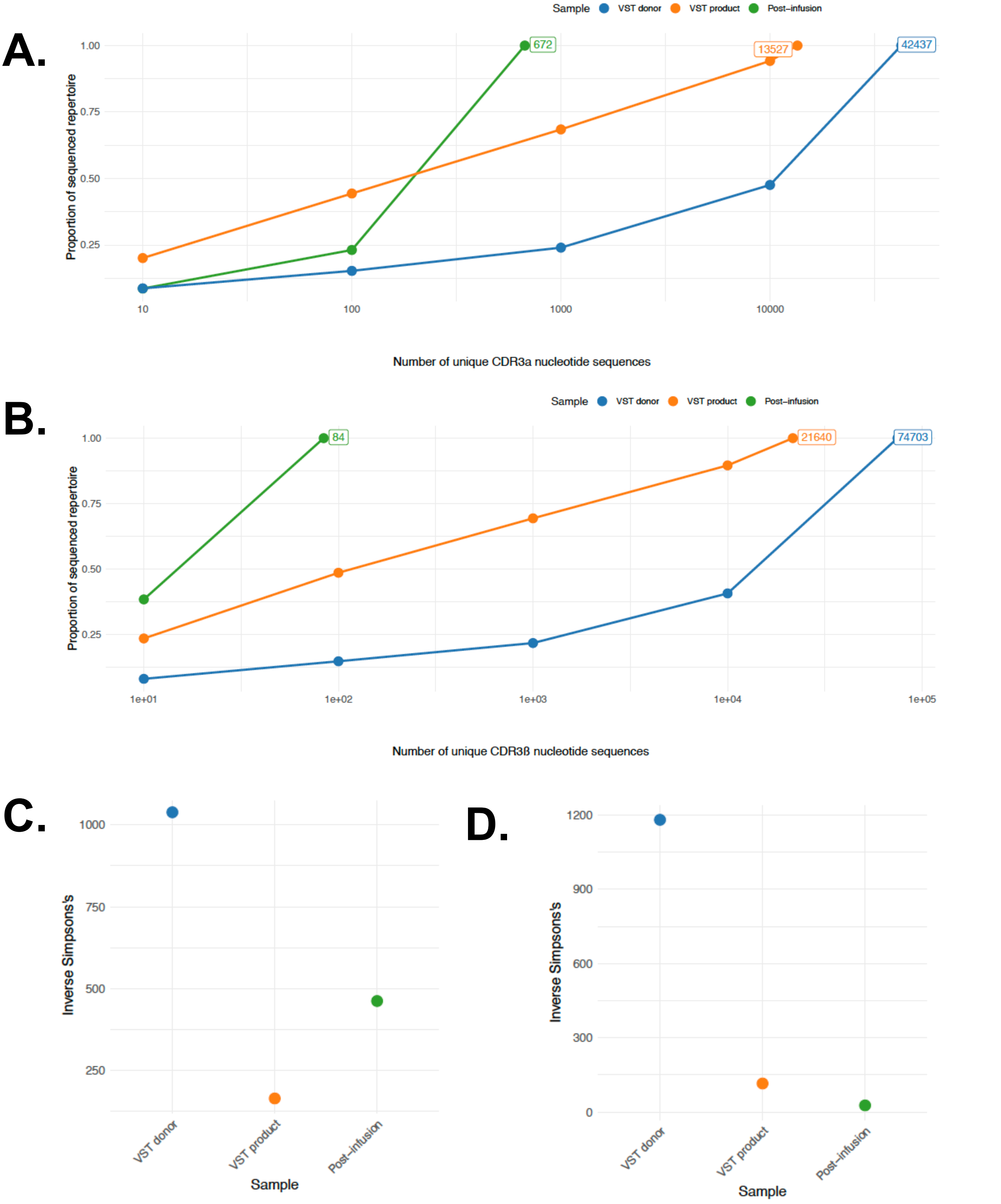

Supplemental Figure 4: Clonotype proportions and diversity of the VST product, VST donor peripheral blood mononuclear cells, and recipient peripheral blood mononuclear cells at day +30. A-B: Proportion of total repertoire versus unique TCRa(A) and TCRb(B) clonotypes. C-D: Inverse Simpson's diversity indices for TCRa (C) and TCRb (D) sequences.

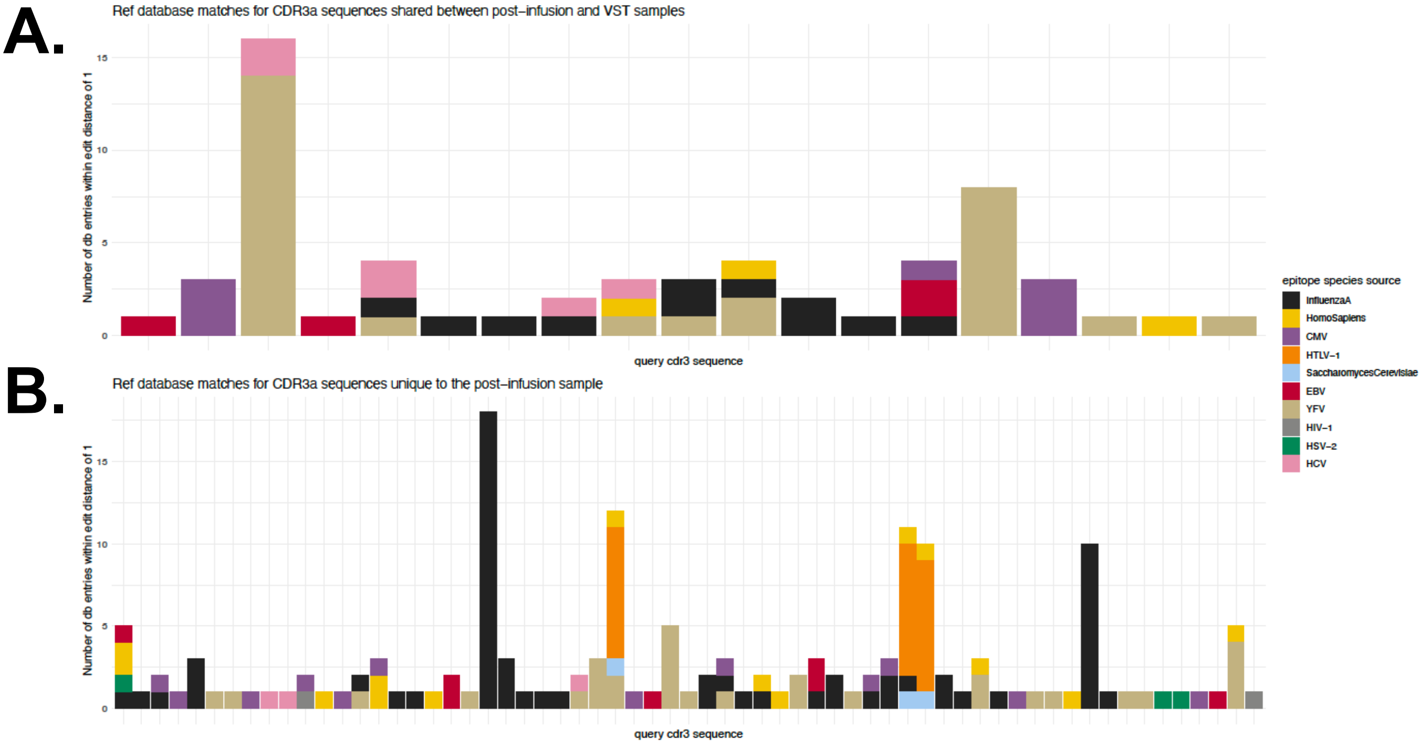

Supplemental Figure 5: Evaluation of public clonotypes in the VST donor and recipient. A. Public clonotype matches for clonotypes shared between the VST donor and recipient (day +30). B. Public clonotype matches with the recipient only at day +30.

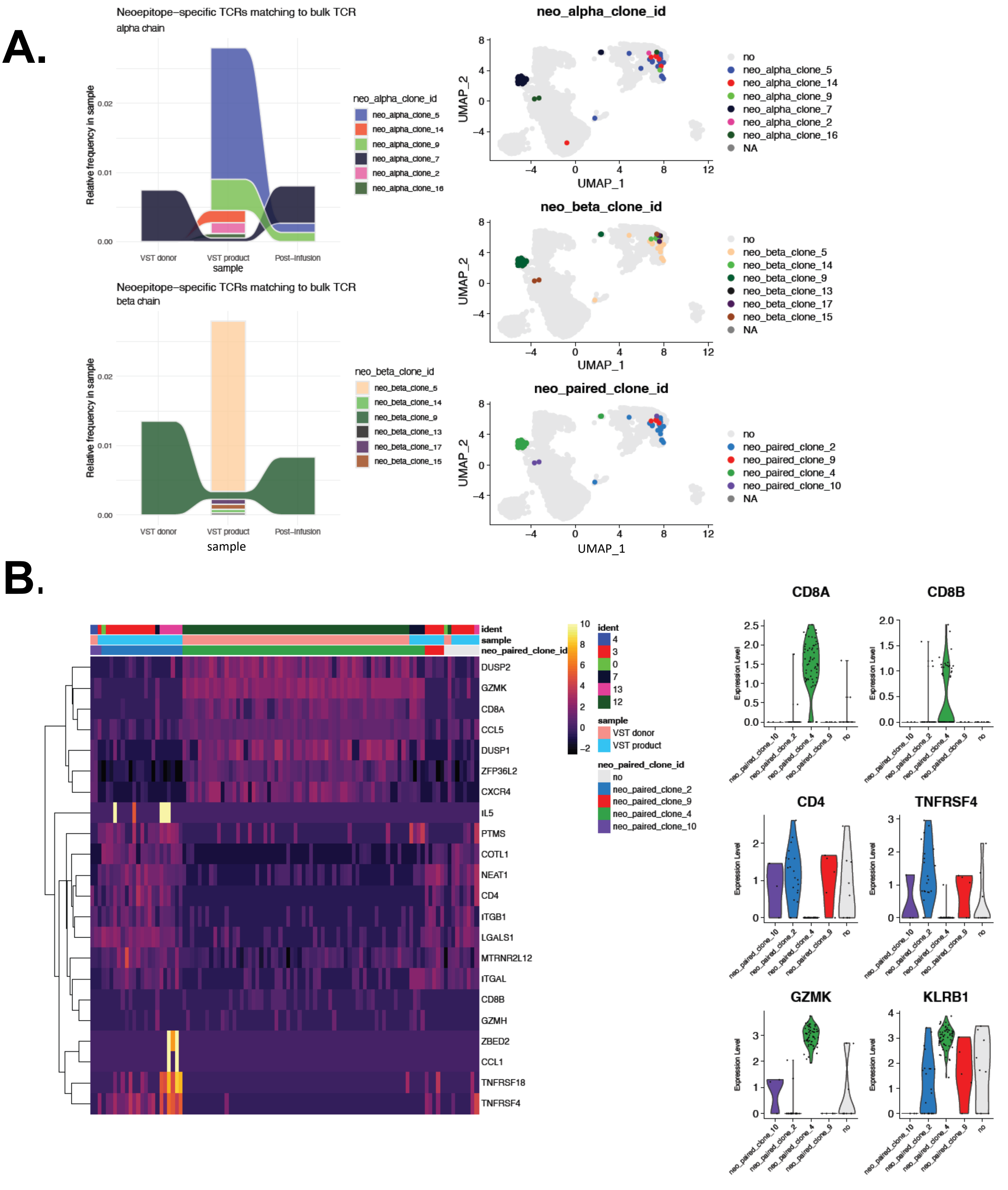

Supplemental Figure 6: Transcriptomic data from neoantigen-specific T cells from the VST donor, product, and recipient. A. Overlap of sorted neoantigen-specific clonotypes in the VST product, donor, and recipient following bulk TCRa (upper) and TCRb (lower) sequencing, and corresponding phenotypes based on principle component analysis. B. Gene expression of sorted neoantigen-specific T cell clones from the VST donor by heat map as well as selected individual genes.

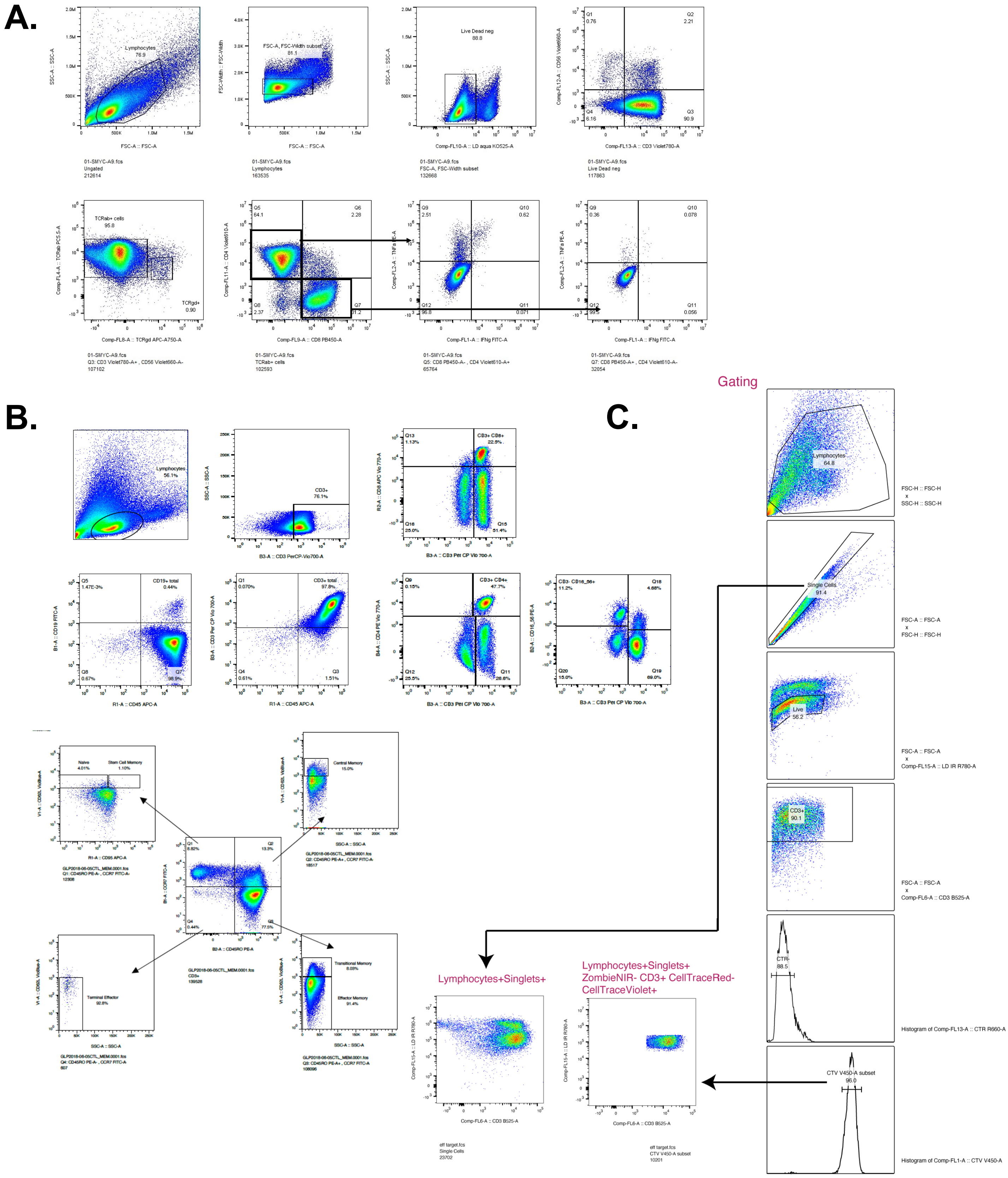

Supplemental Figure 7: Gating strategies for flow cytometry. A. Gating for Intracellular cytokine staining panel (Supplemental Figure 4). B. Gating for surface flow cytometry (Figure 1B, Supplemental Figures 1B and 3A). C. Gating for mixed lymphocyte reactions (Supplemental Figure 2). LD=live/dead, CTR= cell trace red.

Supplemental Table 1: HLA details of the Recipient, BMT donor, VST donor, and mismatched control

	HLA-A	HLA-B	HLA-C	DRB1	DQB1	DPB1
**Recipient**	31:01:00<br>68:01:00	35:01:00<br>xx	03:04<br>07:02	04:07<br>08:02	03:02<br>04:02	04:02<br>14:01
**BMT donor (father)**	02:01:01:73<br>68:01:51	35:01:01:02<br>35:17:01	03:04:01:02<br>04:01:01:01	08:02:01:01<br>14:06:01	03:BJFYP<br>04:BJFYS	04:02:01:02<br>14:01:01:01
**P0230D (VST donor)**	02:11:01:01<br>68:01:02:02	15:18:01:02<br>40:06:01:02	07:04:01:01<br>15:02:01:01	15:01:01:01<br>15:02:01:01	06:01:01:01<br>06:01:01:01	04:01:01:01<br>04:01:01:41
**HLA-mismatched donor (MLR)**	03:01<br>29:03	14:02<br>35:01	04:01<br>08:02	14:54<br>07:01	02:02<br>05:03	04:01<br>15:01

Supplemental Table 2: Candidate Neoantigens based on whole genome sequencing

Chr	Pos	Ref	Variant	Gene	Change	NT / Protein Change	PopFreq Max	VST donor	BMT Donor	Son1	Son2
X	2836181	A	T	ARSD	nonsynonymous_SNV	c.T527A:p.M176K	5.00E-05	absent	het	het	het
X	2836184	C	T	ARSD	nonsynonymous_SNV	c.G524A:p.G175D	0.0001	absent	het	het	het
X	2836211	A	T	ARSD	nonsynonymous_SNV	c.T497A:p.L166Q	0.0002	absent	het	het	het
X	2835863	G	T	ARSD	nonsynonymous_SNV	c.C845A:p.A282D	0.0003	absent	het	het	het
X	2836238	G	A	ARSD	nonsynonymous_SNV	c.C470T:p.S157F	0.0004	absent	het	het	het
X	2836041	A	T	ARSD	nonsynonymous_SNV	c.T667A:p.F223I	0.0005	absent	het	het	het
X	2836047	C	T	ARSD	nonsynonymous_SNV	c.G661A:p.G221S	0.0006	absent	het	het	het
19	36632042	CGGCGGCGGCGGCGGTGGTGGA	C,CGGCGGCGGCGGCGGTGGAGGA	CAPNS1	nonframeshift_deletion	c.130_150del:p.G50_G56del	0.0011	absent	Two alt alleles	het	absent
16	70208251	G	A	CLEC18C	nonsynonymous_SNV	c.G70A:p.A24T	0.0073	absent	het	het	absent
17	62892031	C	T	LRRC37A3	nonsynonymous_SNV	c.G1345A:p.V449I	0.0308	absent	het	absent	het
17	62891989	G	A	LRRC37A3	nonsynonymous_SNV	c.C1387T:p.P463S	0.0323	absent	het	absent	het
1	146400104	G	A	NBPF12	nonsynonymous_SNV	G580A:p.A194T	.	absent	het	absent	het
6	30954245	T	C	MUC21	nonsynonymous_SNV	c.T293C:p.V98A	.	absent	het	het	het
Y	21154426	G	A	CD24	nonsynonymous_SNV	c.C170T:p.A57V	.	absent	het	absent	het
17	62892031	C	T	LRRC37A3	nonsynonymous_SNV	c.G1345A:p.V449I	0.0308	absent	het	het	absent
17	62891989	G	A	LRRC37A3	nonsynonymous_SNV	c.C1387T:p.P463S	0.0323	absent	het	het	absent
17	62892071	G	C	LRRC37A3	nonsynonymous_SNV	c.C1305G:p.H435Q	0.0536	absent	het	het	absent
16	14960480	A	G	NOMO1	nonsynonymous_SNV	c.A1738G:p.M580V	0.0652	absent	het	het	absent
12	44238723	G	A	TMEM117	nonsynonymous_SNV	c.G269A:p.R90H	0.0687	absent	het	het	absent
19	5720074	G	C	LONP1	nonsynonymous_SNV	c.C70G:p.L24V	0.0968	absent	het	het	absent

Supplemental Table 3: Prior use of VST product P0230D

	HLA-A	HLA-B	HLA-C	HLA-DRB1	HLA-DQB1	Degree of match	Viral specificity
**Patient HLA**	23:01, 68:01	15:03, 35:01	02:10, 04:01	03:01, 11:01	02:01, 06:02		
**cord 1**	01:01, 68:01	15:01, 35:03	03:03, 04:01	03:01, 03:01	02:01, 02:01		
**cord 2 (engrafted)**	23:01, 68:02	15:03, 35:01	03:04, 04:01	11:01, 11:01	03:19, 06:02		
**VST products**							
P0231D (1st infusion)	02:01, **68:01**	18:01, 27:05	**02:02,** 07:01	**03:01**, 09:01	**02:01,** 03:03	4/10	CMV: +++A02, +++A68(pp65 and IE1)
P0230D (2nd infusion)	02:11, **68:01**	**15:18**, 40:06	07:04, 15:02	15:01, 15:02	**06:01, 06:01**	3/10	CMV: +++A68 (IE1)
P0108D (3rd infusion)	68:02, 34:02	15:03, 81:01	18:xx, 02:xx	15:03, 16:02	05:xx, 06:02	2/10 (4/10 recip)	CMV: unknown

Supplemental Table 4: Proposed suitability requirements for third-party Immune Effector Therapy Donation

Physical requirements
Donors must be in good health, with no history of recent illness within the past month prior to planned blood donation.
Donors must be ≤ 35 years of age (women) and ≤ 40 years of age (men).
History requirements
Donors shall have no immediate or recent illnesses.
Individuals with any infection (acute or chronic) in the prior month would not be suitable.
Individuals who have received antibiotic or antiviral therapy in the prior month would not be suitable.
Donors shall have minimal risk of cell-mediated allosensitization.
Donors who have received previous blood transfusions or blood products would not be suitable.
Women who have had 2 or more pregnancies would not be suitable.
Donors shall not have had recent exposure to potentially transmissible attenuated viruses.
Those who received any live vaccine in the previous 3 months would not be suitable
Donors who have had close contact with any individual who received the smallpox vaccine in the previous 3 months would not be suitable.
Donors shall not have a history of autoimmune or other immune-mediated disease
Suitable donors must not have history of any of the following conditions:
Immune thrombocytopenic purpura
Autoimmune hemolytic anemia
Hashimoto's thyroiditis
Grave's disease
Inflammatory bowel disease (Crohn's disease or Ulcerative colitis)
Rheumatoid arthritis
Scleroderma
Immune-mediated glomerulonephritis or related kidney disease
Systemic Lupus Erythematosus
Psoriatic arthritis
Celiac disease
Type-I diabetes mellitus
Other immune-mediated diseases which in the opinion of the evaluating physicians, would render the donor unsuitable for IEC donation.
History of psoriasis or vitiligo may not render a donor unsuitable if the disease is quiescent and is not being treated with immunosuppressive therapy.
Donors shall not have a history of moderate to severe atopic conditions
History of any of the following conditions would make a donor unsuitable:
Atopic dermatitis: moderate to severe disease in the prior 5 years
Asthma: moderate to severe persistent asthma (based on NAEPP guidelines) that has been diagnosed or treated in the prior 10 years.
Donors must not have a history of any of the following conditions or procedures:
Hematologic or solid tumors
Solid organ transplant
Hematopoietic stem cell transplantation
Open heart surgery
Elective or emergency surgical procedures in the past 6 months (excluding skin biopsies).
Have, or be a symptomatic carrier of, any form of primary immunodeficiency disorder.
Family members of any patient with a known primary immunodeficiency disorder must undergo immunologic workup prior to potential donation for IEC therapy.
Donors may not have received any of the following medications or therapies
Monoclonal antibody therapies (at any time in the past).
Oral or IV corticosteroid therapy within the past 3 months
High potency topical steroids (class I-II) within the past 3 months.
Other immunosuppressive therapy, including cyclosporin, sirolimus, tacrolimus, ruxolitinib, tofacitinib, methotrexate, 6MP, azathioprine, dapsone, or other related therapies (at any time in the past)

Supplemental Table 5: Supplemental Recipient Eligibility requirements for VST infusion

Recipient Supplemental Eligibility Requirements
Patients who have undergone HSCT must have stable chimerism (defined as >95% donor chimerism [CD33 or whole blood chimerism], OR >90% donor chimerism [CD33 or whole blood] with <5% change between subsequent tests separated by at least 1 week).
Patients with severe combined immunodeficiency who are less than 100 days post α/β TCR depleted HSCT would be ineligible for VST infusion.

Supplemental Table 6: Surface stain antibody panel

Tube	Antibodies										
Channel	Violet 660	APC-A750	K0525	FITC	PE	PC7	APC	PB450	Violet610	PerCP PC5	ECD mCherry	APC-A700
Color	BV650	APC Vio770	Vio-Green	FITC	PE	PEVio-700	APC	VioBlue	LD yellow	PerCP Vio	PEVio-615	Alexa-700
Isotype<br><br>Per tube: 25ul	CD56<br><br>(5ul)	CD3<br><br>(2ul)	CD45<br><br>(2ul)	REA<br><br>(2ul)	REA (2ul)	Mouse IgG1<br><br>(2ul)	REA<br><br>(2ul)	IgG1k<br><br>(2ul)	LD yellow<br><br>(2ul/ml)	REA<br><br>(2ul)	REA<br><br>(2ul)	mouse IgG1,K<br><br>(2ul)
NK Panel<br><br>Per tube: 31ul+HLA	CD56<br><br>(5ul)	CD3<br><br>(2ul)	CD45<br><br>(2ul)	CD52<br><br>(2ul)	HLA<br><br>-<br>(varies)	NKG2D<br><br>(10ul)	CD57<br><br>(2ul)	CD20<br><br>(2ul)	LD yellow<br><br>(2ul/ml)	CD8<br><br>(2ul)	iNKT<br><br>(2ul)	CD4<br><br>(2ul)

Supplemental Table 7: Intracellular staining antibody panel

**Tube**	**Antibodies**									
Channel	**PB450**	**KO525**	**BV610**	**BV660**	**BV780**	**FITC**	**PE**	**ECD**	**APC**	**APC-A700**
Color	BV421	Ghost 510	BV605	BV750	BV785			PE Dazzle	APC	Alexa 700
Ab	CD8	LD	CD4	CD56	CD3	HLA	TNFa	CD45RO	IFNg	CCR7

Supplemental Table 8: Product release surface antibody panel

PanLeuko	TCR	DC
CD14 VioBlue		CD14 VioBlue
CD19 FITC	TCRab FITC	HLA DR FITC
CD16/CD56 PE	TCRgd PE	CD83 PE
CD3 Per CP Vio 700	CD3 Per CP Vio 700	CD3 Per CP Vio 700
CD4 PE Vio 770	CD4 PE Vio 770	
CD45 APC	CD45 APC	CD45 APC
CD8 APV Vio770	CD8 APV Vio770	

Supplemental Table 9: Antibodies Details: Intracellular flow panel

Antibody	Manufacturer	Catalog #	Clone	Format	Vol/ test (1E6 cells)	Mfg references
Brilliant Violet 421™ anti-human CD8a Antibody	BioLegend	301036	RPA-T8	BV421	5ul	https://www.biolegend.com/en-us/products/brilliant-violet-421-anti-human-cd8a-antibody-7152
LIVE/DEAD™ Fixable Aqua Dead Cell Stain Kit, for 405 nm excitation	Invitrogen	L34966	N/A	Aqua	1ul/ml	https://www.thermofisher.com/document-connect/document-connect.html?url=https://assets.thermofisher.com/TFS-Assets%2FLSG%2Fmanuals%2Flive_dead_fixable_dead_cell_stains_man.pdf
Brilliant Violet 605™ anti-human CD4 Antibody	BioLegend	317438	OKT4	BV605	5ul	https://www.biolegend.com/en-us/products/brilliant-violet-605-anti-human-cd4-antibody-7820
Brilliant Violet 650™ anti-human CD56 (NCAM) Antibody	BioLegend	318344	HCD56	BV650	5ul	https://www.biolegend.com/en-us/products/brilliant-violet-650-anti-human-cd56-ncam-antibody-8780
Brilliant Violet 785™ anti-human CD3 Antibody	BioLegend	317330	OKT3	BV785	5ul	https://www.biolegend.com/en-us/products/brilliant-violet-785-anti-human-cd3-antibody-7977
PE anti-human TNF-α Antibody	BioLegend	502909	MAb11	PE	5ul	https://www.biolegend.com/en-us/products/pe-anti-human-tnf-alpha-antibody-1346
PE/Dazzle™ 594 anti-human CD45RO Antibody	BioLegend	304248	UCHL1	PE/Dazzle™ 594	5ul	https://www.biolegend.com/en-us/products/pe-dazzle-594-anti-human-cd45ro-antibody-12489
PerCP/Cyanine5.5 anti-human TCR α/β Antibody	BioLegend	306724	IP26	PerCP/Cyanine5.5	5ul	https://www.biolegend.com/en-us/products/percp-cyanine5-5-anti-human-tcr-alpha-beta-antibody-9232
PE/Cyanine7 anti-human CD107a (LAMP-1) Antibody	BioLegend	328618	H4A3	PE/Cyanine7	5ul	https://www.biolegend.com/en-us/products/pe-cyanine7-anti-human-cd107a-lamp-1-antibody-7707
APC anti-human IFN-γ Antibody	BioLegend	502512	4S.B3	APC	5ul	https://www.biolegend.com/en-us/products/apc-anti-human-ifn-gamma-antibody-1012
Alexa Fluor® 700 anti-human CD197 (CCR7) Antibody	BioLegend	353244	G043H7	AF700	5ul	https://www.biolegend.com/en-us/products/alexa-fluor-700-anti-human-cd197-ccr7-antibody-13407
APC/Fire™ 750 anti-human TCR γ/δ Antibody	BioLegend	331228	B1	APC/Fire™ 750	5ul	https://www.biolegend.com/en-us/products/apc-fire-750-anti-human-tcr-gamma-delta-antibody-14101

Supplemental Table 10: HLA Antibody details

HLA Specificity	Manufacturer	Splits and Associated Ab	Catalog #	Clone	Channels	Volume used/cells	Mfg references
**A2**	Milteyni	A203#, A210#	130-118-969	REA517	FITC	2 ul / 200k cells	https://www.miltenyibiotec.com/US-en/products/hla-a2-antibody-anti-human-reafinity-rea517.html#conjugate=fitc:size=100-tests-in-200-ul
**A3**	Milteyni	N/A	130-115-739	REA950	FITC	2 ul / 200k cells	https://www.miltenyibiotec.com/US-en/products/hla-a3-antibody-anti-human-reafinity-rea950.html#conjugate=fitc:size=100-tests-in-200-ul
**A28:A2**	Milteyni	A68, A69	130-099-601	REA142	FITC	10 ul / 200k cells	https://www.miltenyibiotec.com/US-en/products/hla-a2-a28-antibody-anti-human-reafinity-rea142.html#conjugate=fitc:size=100-tests-in-200-ul
**A9**	Milteyni	A23, A24, A2403#	130-099-524	REA127	FITC	2 ul / 200k cells	https://www.miltenyibiotec.com/US-en/products/hla-a9-antibody-anti-human-reafinity-rea127.html#conjugate=fitc:size=100-tests-in-1-ml
**A30:A31**	One Lambda	N/A	Steptavidin FITC: 405201	Not appliable	Biotin (+ Streptavidin FITC and PE conjugates)	2ul/200k cells	N/A
**B7**	Thermo(FITC)	B703#	MA1-82180	BB7.1	FITC	10 ul / 200k cells	https://www.thermofisher.com/antibody/product/HLA-B7-Antibody-clone-BB7-1-Monoclonal/MA1-82180
**B12**	Milteyni	B44,B45	130-099-862	REA138	FITC	30 tests in 300ul	https://www.miltenyibiotec.com/US-en/products/hla-b12-antibody-anti-human-reafinity-rea138.html#conjugate=fitc:size=30-tests-in-300-ul
**Bw4**	Milteyni	B5, B5102, B5103, B13, B17, B27, B37, B38(16), B44(12), B47, B49(21), B51(5), B52(5), B53, B57(17), B58(17), B59, B63(15), B77(15) A9, A23(9), A24(9), A2403, A25(10), A32(19)	130-103-846	REA274	FITC	10ul/200k cells	https://www.miltenyibiotec.com/US-en/products/hla-class-i-bw4-antibody-anti-human-reafinity-rea274.html#conjugate=pe:size=30-tests-in-60-ul
**Bw6**	Milteyni	B7, B703, B8, B14, B18, B22, B2708, B35, B39(16), B3901, B3902, B40, B4005, B41, B42, B45(12), B46, B48, B50(21),	130-123-264	REA143	FITC	2 ul / 200k cells	https://www.miltenyibiotec.com/US-en/products/hla-class-i-bw6-antibody-anti-human-reafinity-rea143.html#conjugate=fitc:size=100-tests-in-200-ul

		B54(22), B55(22), B56(22), B60(40), B61(40), B62(15), B64(14), B65(14), B67, B70, B71(70), B72(70), B73, B75(15), B76(15), B78, B81, B82					
B7, B27	Miltenyi	B27 splits to B2708#	130-120-234	REA176	FITC	2 ul / 200k cells	https://www.miltenyibiotec.com/US-en/products/hla-b7-b27-antibody-anti-human-reafinity-rea176.html#conjugate=vio-bright-fitc:size=100-tests-in-200-ul
			130-120-308	REA176	FITC	2 ul / 1E6 cells	https://www.miltenyibiotec.com/US-en/products/hla-b7-b27-antibody-anti-human-reafinity-rea176.html#conjugate=vio-bright-fitc:size=30-tests-in-60-ul
HLA_A33, B8	Life Span Biosci	N/A	LS-C24464-100	Not appliable	unconjugated	Use as manufacturer's guide	https://www.lsbio.com/antibodies/hla-a33b8-antibody-ls-c24464/25046
Anti-HLA-A24 (Human) mAb	MBL international corp.	A2403#	K0208-4 & K0208-5	17A10	FITC	Use as manufacturer's guide	https://www.mblbio.com/bio/g/dtl/A/?pcd=K0208-5

Supplemental Table 11: Product release surface staining panel

Antibody/fluorophore	Vendor	Catalog Number	Clone	Volume Used/2E5 cells	**Mfg references**
CD45 APC	Miltenyi	130-110-633	REA747	1ul	https://www.miltenyibiotec.com/US-en/products/cd45-antibody-anti-human-reafinity-rea747.html#conjugate=apc:size=100-tests-in-200-ul
CD3 PerCP Vio 700	Miltenyi	130-113-141	REA613	1ul	https://www.miltenyibiotec.com/US-en/products/cd3-antibody-anti-human-reafinity-rea613.html#conjugate=percp-vio-700:size=100-tests-in-200-ul
CD19 FITC	Miltenyi	130-113-645	REA675	1ul	https://www.miltenyibiotec.com/US-en/products/cd19-antibody-anti-human-reafinity-rea675.html#conjugate=fitc:size=100-tests-in-200-ul
CD14 VioBlue	Miltenyi	130-110-524	REA599	2ul	https://www.miltenyibiotec.com/US-en/products/cd14-antibody-anti-human-reafinity-rea599.html#conjugate=vioblue:size=100-tests-in-200-ul
CD4 PE Vio770	Miltenyi	130-113-227	REA623	1ul	https://www.miltenyibiotec.com/US-en/products/cd4-antibody-anti-human-reafinity-rea623.html#conjugate=pe-vio-770:size=100-tests-in-200-ul
CD8 APC Vio770	Miltenyi	130-110-681	REA734	1ul	https://www.miltenyibiotec.com/US-en/products/cd8-antibody-anti-human-reafinity-rea734.html#conjugate=apc-vio-770:size=100-tests-in-200-ul
CD16 PE	Miltenyi	130-113-393	REA423	1ul	https://www.miltenyibiotec.com/US-en/products/cd16-antibody-anti-human-reafinity-rea423.html#conjugate=pe:size=100-tests-in-200-ul
CD 56 PE	Miltenyi	130-113-312	REA196	1ul	https://www.miltenyibiotec.com/US-en/products/cd56-antibody-anti-human-reafinity-rea196.html#conjugate=pe:size=100-tests-in-200-ul
CD83 PE	Miltenyi	130-110-503	REA714	1ul	https://www.miltenyibiotec.com/US-en/products/cd83-antibody-anti-human-reafinity-rea714.html#conjugate=pe:size=100-tests-in-200-ul
TCRab FITC	Miltenyi	130-113-538	RES652	1ul	https://www.miltenyibiotec.com/US-en/products/tcra-b-antibody-anti-human-reafinity-rea652.html#conjugate=fitc:size=100-tests-in-200-ul
TCRgd PE	Miltenyi	130-113-512	REA591	1ul	https://www.miltenyibiotec.com/US-en/products/tcrg-d-antibody-anti-human-reafinity-rea591.html#conjugate=pe:size=100-tests-in-200-ul
CD45RO PE	Miltenyi	130-113-559	REA611	2ul	https://www.miltenyibiotec.com/US-en/products/tcrg-d-antibody-anti-human-reafinity-rea591.html#conjugate=pe:size=100-tests-in-200-ul
CCR7 FITC	Miltenyi	130-120-468	REA546	2ul	https://www.miltenyibiotec.com/US-en/products/cd197-ccr7-antibody-anti-human-reafinity-rea546.html#conjugate=fitc:size=100-tests-in-200-ul
CD95 APC	BD Bio	558814	DX2	2ul	https://www.bdbiosciences.com/en-us/products/reagents/flow-cytometry-reagents/research-reagents/single-color-antibodies-ruo/apc-mouse-anti-human-cd95.558814
CD62 VioBlue	Miltenyi	130-113-622	145/15	2ul	https://www.miltenyibiotec.com/US-en/products/cd62l-antibody-anti-human-145-15.html#conjugate=vioblue:size=100-tests-in-200-ul
HLA DR FITC	Miltenyi	30-111-788	REA805	1ul	https://www.miltenyibiotec.com/US-en/products/hla-dr-antibody-anti-human-reafinity-rea805.html#conjugate=vio-bright-r720:size=100-tests-in-200-ul
FcR Blocking Reagent	Miltenyi	130-059-901	N/A	10ul	https://www.miltenyibiotec.com/US-en/products/fcr-blocking-reagent-human.html#130-059-901

Supplemental Table 12: Pentamer details

Product description	**Manufacturer**	Cat#	**Vol/test (1E6 cells)**	Mfr reference
F395-0A-D - 395 - A*02:01 - YIGEVLVSV - Pentamer - Unlabeled	Proimmune	F395-0A-D	2ul	https://www.proimmune.com/list-of-catalog-pentamers/
F473-0A-D - 473 - A*02:01 - VLHDDLLEA - Pentamer - Unlabeled	Proimmune	F473-0A-D	2ul	https://www.proimmune.com/list-of-catalog-pentamers/
F581-0A-D - 581 - A*02:01 - RTLDKVLEV - Pentamer - Unlabeled	Proimmune	F581-0A-D	2ul	https://www.proimmune.com/list-of-catalog-pentamers/
F175-0A-D - 175 - A*02:01 - FIDSYICQV - Pentamer - Unlabeled	Proimmune	F175-0A-D	2ul	https://www.proimmune.com/list-of-catalog-pentamers/
K4B-D - Fluorotag - 150 test APC	Proimmune	K4B-D	8ul	https://www.proimmune.com/ecommerce/pdf_files/PR06U.pdf

Supplemental Table 13: Mixed Lymphocyte Reaction Celltrace details

Item	Cat#	Mfr reference
CellTrace™ Far Red Cell Proliferation Kit, for flow cytometry	**C34564**	https://www.thermofisher.com/order/catalog/product/C34564
CellTrace™ Violet Cell Proliferation Kit, for flow cytometry	**C34557**	https://www.thermofisher.com/order/catalog/product/C34557
CellTrace™ Far Red Cell Proliferation Kit, for flow cytometry	**C34564**	https://www.thermofisher.com/order/catalog/product/C34564

User Guide : https://www.thermofisher.com/document-connect/document-connect.html?url=https://assets.thermofisher.com/TFS-Assets%2FLSG%2Fmanuals%2FMAN0002595_CellTrace_Cell_Proliferation_Kits_UG.pdf
