# Supplementary material for: Secondary bone marrow graft loss after third-party virus-specific T cell infusion: Case report of a rare complication
Source: Nat Commun. 2024 Mar 29;15:2749. doi: 10.1038/s41467-024-47056-3 (PMC10980733; doi:10.1038/s41467-024-47056-3)
Supplement: Supplementary file 3 — Description of Additional Supplementary Files [file 41467_2024_47056_MOESM3_ESM.pdf]

Description of Additional Supplementary Files

Supplementary Data 1: Sequences of utilized peptide libraries based on previously published XY and neoantigens.

Supplementary Data 2: Neoantigen sequences utilized based on genome sequencing of P0230D and family.

Supplementary Data 3: T cell receptor sequencing results from patient VST donor. Samples from VST donor (P0230D) were sorted to isolate CD3+/IFN-g+ cells following peptide library restimulation, and sequenced alongside bulk, unstimulated cells from the same samples and time points.   VST donor PBMC (from time of VST expansion) or VST product were utilized for sequencing. Recipient peripheral blood mononuclear cells from day +30 post-VST infusion were utilized.
