# Supplementary material for: Secondary bone marrow graft loss after third-party virus-specific T cell infusion: Case report of a rare complication
Source: Nat Commun. 2024 Mar 29;15:2749. doi: 10.1038/s41467-024-47056-3 (PMC10980733; doi:10.1038/s41467-024-47056-3)
Supplement: Supplementary file 7 — Reporting Summary [file 41467_2024_47056_MOESM7_ESM.pdf]

nature portfolio

Corresponding author(s): Catherine Bollard

Last updated by author(s): 10/23/2023

Reporting Summary

Nature Portfolio wishes to improve the reproducibility of the work that we publish. This form provides structure for consistency and transparency in reporting. For further information on Nature Portfolio policies, see our Editorial Policies and the Editorial Policy Checklist.

Statistics

For all statistical analyses, confirm that the following items are present in the figure legend, table legend, main text, or Methods section.

n/a	Confirmed	
☐	☒	The exact sample size (*n*) for each experimental group/condition, given as a discrete number and unit of measurement
☐	☒	A statement on whether measurements were taken from distinct samples or whether the same sample was measured repeatedly
☒	☐	The statistical test(s) used AND whether they are one- or two-sided<br>*Only common tests should be described solely by name; describe more complex techniques in the Methods section.*
☐	☒	A description of all covariates tested
☐	☒	A description of any assumptions or corrections, such as tests of normality and adjustment for multiple comparisons
☐	☒	A full description of the statistical parameters including central tendency (e.g. means) or other basic estimates (e.g. regression coefficient) AND variation (e.g. standard deviation) or associated estimates of uncertainty (e.g. confidence intervals)
☒	☐	For null hypothesis testing, the test statistic (e.g. *F*, *t*, *r*) with confidence intervals, effect sizes, degrees of freedom and *P* value noted<br>*Give P values as exact values whenever suitable.*
☒	☐	For Bayesian analysis, information on the choice of priors and Markov chain Monte Carlo settings
☐	☒	For hierarchical and complex designs, identification of the appropriate level for tests and full reporting of outcomes
☒	☐	Estimates of effect sizes (e.g. Cohen's *d*, Pearson's *r*), indicating how they were calculated

Our web collection on statistics for biologists contains articles on many of the points above.

Software and code

Policy information about availability of computer code

Data collection	Whole genome sequencing: DNA sequencing reads were aligned to human genome build UCSC hg19 (hs37d5) in BaseSpace Sequence Hub using DRAGEN Germline Pipeline (v3.4.5). Variant calls files were annotated using annovar (v2020-06-08) with refseq gene function annotations and gnomad allele frequency (population max). Annotated vcf files were filtered using bcftools (v1.12).<br>Chimerism: Final chimerism determinations were performed using ChimeRMarker™ Genetic Analysis software (SoftGenetics, State College, PA. USA).<br>Cytokine assays: All plasma samples were aquired on a MAGPIX system (Luminex, Austin, TX).<br>Flow cytometry: Samples were acquired on a CytoFLEX cytometer (Beckman Coulter, Brea, CA), and sorted samples were run on a CytoFlex SRT Cell Sorter (Beckman Coulter, Brea, CA).<br>Single cell RNASeq and TCR sequencing: Both gene expression and TCR libraries were sequenced on a NovaSeq 6000 (Illumina, San Diego, CA) Individually sequenced gene expression libraries were aggregated using CellRanger (v3.1.0) with default parameters.<br>ELISpot results were obtained using a KS Elispot Reader (Carl Zeiss, Inc., Thornwood, NY), Software Version 5.9.16.
Data analysis	Flow cytometry: Flow cytometry data was analyzed with FlowJo X (FlowJo LLC, Ashland, OR).<br>Single cell sequencing: 10x data was analyzed using the Seurat package (v 4.1.0) for R (v 4.1.2). Immunarch (v0.6.6) package for R was used to measure TCR sample diversity, clonality, and overlap. VDJ junction mapping and clonotype assembly and annotation using the assembled contigs from migec was done with mixcr (v3.0.13) using the analyze amplicon routine.

For manuscripts utilizing custom algorithms or software that are central to the research but not yet described in published literature, software must be made available to editors and reviewers. We strongly encourage code deposition in a community repository (e.g. GitHub). See the Nature Portfolio guidelines for submitting code & software for further information.

Data

Policy information about availability of data

All manuscripts must include a data availability statement. This statement should provide the following information, where applicable:

- Accession codes, unique identifiers, or web links for publicly available datasets
- A description of any restrictions on data availability
- For clinical datasets or third party data, please ensure that the statement adheres to our policy

The datasets generated during and/or analyzed during the current study have been enclosed in supplemental data. These flow cytometry and single cell sequencing data sets are available on Zenodo under the following link: https://doi.org/10.5281/zenodo.10028505.
Single cell sequencing data is also accessible under Genbank (Bioproject PRJNA1051284)
All remaining data is enclosed within the article, supplementary information, and source data files.

Research involving human participants, their data, or biological material

Policy information about studies with human participants or human data. See also policy information about sex, gender (identity/presentation), and sexual orientation and race, ethnicity and racism.

Reporting on sex and gender	This study involved evaluation of a female subject, the male bone marrow donor, a female T cell donor, and two male family members of the female T cell donor. All subjects provided informed consent for participation in this study. Studies of H-Y specific antigens may have gender-specific impact on immunologic risks in transplantation. All other studies performed should not be impacted by sex and gender.
Reporting on race, ethnicity, or other socially relevant groupings	This is a study of a rare adverse event that occurred in a single infant with severe combined immunodeficiency. We do not report on race/ethnicity or other socially relevant groupings.
Population characteristics	This is a study of an infant with severe combined immunodeficiency (female, age 6 months), her adult bone marrow transplant donor (male, age 27 years), and the utilized adult T cell donor (female, age 47 years).
Recruitment	The subject was recruited on the ACES study (NCT03475212). Eligibility criteria for this study were: patients with primary immunodeficiency or recipients of allogeneic hematopoietic stem cell transplantation with refractory infection with CMV, EBV, or adenovirus, and no other uncontrolled infections, high grade graft versus host disease, or relapse of malignancy. As a pediatric study, subject were limited to those 0-25 years of age. Patients of all genders and ethnicities were eligible for enrollment, with actual subject enrollment limited only by the patient demographics of the 22 centers participating in this study. The study protocol is attached in Supplemental Data.
Ethics oversight	This study was approved at institutional review boards of participating centers (Children's Hospital of Los Angeles, Children's National Hospital, St Jude's Research Children's Hospital, and Children's Hospital of Atlanta) as well as the drug safety monitoring board of the Pediatric Transplantation and Cellular Therapy Consortium (PTCTC).

Note that full information on the approval of the study protocol must also be provided in the manuscript.

Field-specific reporting

Please select the one below that is the best fit for your research. If you are not sure, read the appropriate sections before making your selection.

☒ Life sciences ☐ Behavioural & social sciences ☐ Ecological, evolutionary & environmental sciences

For a reference copy of the document with all sections, see nature.com/documents/nr-reporting-summary-flat.pdf

Life sciences study design

All studies must disclose on these points even when the disclosure is negative.

Sample size	The assays performed in this study were primary descriptive in nature as they pertain to only the patient, her BMT donor, and the adult virus-specific T cell (VST) donor. As such, no sample size calculations could be performed, they were limited to available samples from the study patient, BMT donor, and VST donor, all of which were utilized as detailed in the manuscript.
Data exclusions	No data was excluded from this manuscript.
Replication	All immunoassays were performed in replicate (triplicate where cells allowed) in order to ensure result accuracy. All replicates were successful, and raw data is included in the manuscript appendices. Single cell sequencing was performed on very limited subjects samples, and therefore replication was not possible for these assays.
Randomization	As a single-subject analysis, randomization was not performed.

Blinding	Blinding of results was not possible in these studies given the nature of the assays and the small number of research subjects relevant to this case report. However, subject response data for this trial was reviewed by a blinded group of evaluators across several institutions in order to ensure uniformity in the evaluation of clinical antiviral responses.

Reporting for specific materials, systems and methods

We require information from authors about some types of materials, experimental systems and methods used in many studies. Here, indicate whether each material, system or method listed is relevant to your study. If you are not sure if a list item applies to your research, read the appropriate section before selecting a response.

Materials & experimental systems

n/a	Involved in the study	
☐	☒	Antibodies
☒	☐	Eukaryotic cell lines
☒	☐	Palaeontology and archaeology
☒	☐	Animals and other organisms
☐	☒	Clinical data
☒	☐	Dual use research of concern
☒	☐	Plants

Methods

n/a	Involved in the study	
☒	☐	ChIP-seq
☐	☒	Flow cytometry
☒	☐	MRI-based neuroimaging

Antibodies

Antibodies used

All antibody panels are listed in supplemental data.
Intracellular cytokine panel:
Brilliant Violet 421™ anti-human CD8a Antibody/ BioLegend, #301036, RPA-T8, BV421, 5ul/1E6 cells
LIVE/DEAD Fixable Aqua Dead Cell Stain Kit, for 405 nm excitation, Invitrogen, L34966, Aqua, 1ul/ml
Brilliant Violet 605 anti-human CD4 Antibody, BioLegend, #317438, OKT4, BV605, 5ul/1E6 cells
Brilliant Violet 650 anti-human CD56 (NCAM) Antibody BioLegend, #318344, HCD56, BV650, 5ul/1E6 cells
Brilliant Violet 785 anti-human CD3 Antibody BioLegend, #317330, OKT3, BV785, 5ul/1E6 cells
PE anti-human TNF-α Antibody, BioLegend, #502909, MAb11, PE, 5ul/1E6 cells
PE/Dazzle 594 anti-human CD45RO Antibody, BioLegend, #304248, UCHL1, PE/Dazzle 594, 5ul/1E6 cells
PerCP/Cyanine5.5 anti-human TCR α/β Antibody, BioLegend, #306724, IP26, PerCP/Cyanine5.5, 5ul/1E6 cells
PE/Cyanine7 anti-human CD107a (LAMP-1) Antibody, BioLegend, #328618, H4A3, PE/Cyanine7 5ul/1E6 cells
APC anti-human IFN-γ Antibody, BioLegend, #502512, 4S.B3, APC, 5ul/1E6 cells
Alexa Fluor 700 anti-human CD197 (CCR7) Antibody, BioLegend, #353244, G043H7, AF700, 5ul/1E6 cells
APC/Fire 750 anti-human TCR γ/δ Antibody, BioLegend, #331228, B1, APC/Fire 750, 5ul/1E6 cells

Anti-HLA antibodies:
A2, Milteyni, 130-118-969, REA517 FITC 2 ul / 200k cells
A3, Milteyni 130-115-739, REA950 FITC 2 ul / 200k cells
A28:A2, Milteyni A68, A69 130-099-601, REA142 FITC 10 ul / 200k cells
A9, Milteyni, 130-099-524, REA127" FITC 2 ul / 200k cells
A30:A31, One Lambda N/A Steptavidin FITC: 405201 Biotin (+ Streptavidin FITC and PE conjugates) 2ul/200k cells
B7, Thermo(FITC) B703# MA1-82180, BB7.1 FITC 10 ul / 200k cells
B12, Milteyni, 130-099-862, REA138 FITC 30 tests in 300ul
Bw4, Milteyni, 130-103-846, REA274 FITC 10ul/200k cells
Bw6 Milteyni, 130-123-264 REA143 FITC 2 ul / 200k cells
B7, B27 Miltenyi, 130-120-234 REA176 FITC 2 ul / 200k cells

Release flow cytometry panel:
CD45 APC Miltenyi, 130-110-633 REA747 1ul/2E5 cells
CD3 PerCP Vio 700 Miltenyi, 130-113-141 REA613 1ul/2E5 cells
CD19 FITC Miltenyi, 130-113-645 REA675 1ul/2E5 cells
CD14 VioBlue Miltenyi, 130-110-524 REA599 2ul/2E5 cells
CD4 PE Vio770 Miltenyi, 130-113-227 REA623 1ul/2E5 cells
CD8 APC Vio770 Miltenyi, 130-110-681 REA734 1ul/2E5 cells
CD16 PE Miltenyi, 130-113-393 REA423 1ul/2E5 cells
CD 56 PE Miltenyi, 130-113-312 REA196 1ul/2E5 cells
CD83 PE Miltenyi, 130-110-503 REA714 1ul/2E5 cells
TCRab FITC Miltenyi, 130-113-538 RES652 1ul/2E5 cells
TCRgd PE Miltenyi, 130-113-512 REA591 1ul/2E5 cells
CD45RO PE Miltenyi, 130-113-559 REA611 2ul/2E5 cells
CCR7 FITC Miltenyi, 130-120-468 REA546 2ul/2E5 cells
CD95 APC BD Bio, 558814 DX2 2ul/2E5 cells

	CD62 VioBlue Miltenyi, 130-113-622 145/15 2ul/2E5 cells<br>HLA DR FITC Miltenyi, 30-111-788 REA805 1ul/2E5 cells<br><br>Pentamers:<br>F395-0A-D - 395, A*02:01 - YIGEVLVSV - Pentamer -Unlabeled, Proimmune, #F395-0A-D, 2ul/1E6 cells<br>F473-0A-D - 473, A*02:01 - VLHDDLLEA - Pentamer - Unlabeled, Proimmune #F473-0A-D, 2ul/1E6 cells<br>F581-0A-D - 581, A*02:01 - RTLDKVLEV - Pentamer - Unlabeled, Proimmune #F581-0A-D, 2ul/1E6 cells<br>F175-0A-D - 175, A*02:01 - FIDSYICQV - Pentamer - Unlabeled, Proimmune #F175-0A-D, 2ul/1E6 cells
Validation	Anti-cytokine antibodies underwent titration to establish optimal staining index for human leukocytes as listed above. All other antibodies were utilized per manufacturer recommendations. All antibody dilutions are listed above and in the supplemental data.

Clinical data

Policy information about clinical studies

All manuscripts should comply with the ICMJEguidelines for publication of clinical research and a completedCONSORT checklist must be included with all submissions.

Clinical trial registration	NCT03475212
Study protocol	The full clinical protocol of the ACES trial is attached in the Supplemental Data.
Data collection	Data from the subjects was collected by investigators at the primary clinical site (Children's Hospital of Atlanta) between September-December of 2019. Subsequent data was generated at Children's National Hospital and St Jude hospital from research samples between November 2019-August 2022.
Outcomes	The primary endpoints of this study were safety of T cell infusion, efficacy against targeted viruses based on blood viral PCR at 1 month post infusion, and feasibility of third-party T cell infusion (ability to identify a suitable banked product). Secondary endpoints were immune reconstitution against targeted viruses, persistence of infused cells, and overall survival at 1 year post infusion. These outcomes were assessed by investigators at each study site via clinical lab testing as well as correlative assays in blood performed centrally at Children's National Hospital.

Flow Cytometry

Plots

Confirm that:

- [x] The axis labels state the marker and fluorochrome used (e.g. CD4-FITC).
- [x] The axis scales are clearly visible. Include numbers along axes only for bottom left plot of group (a 'group' is an analysis of identical markers).
- [x] All plots are contour plots with outliers or pseudocolor plots.
- [x] A numerical value for number of cells or percentage (with statistics) is provided.

Methodology

Sample preparation	All cells were obtained from whole blood via Ficoll centrifugation.
Instrument	All samples were run on a Cytoflex Cytometry (Beckman Coulter) or CytoFlex SRT Cell Sorter
Software	Data was analyzed with FlowJo X (FlowJo LLC, Ashland, OR).
Cell population abundance	For this study, flow cytometry was used to identify antigen-specific T cells targeting CMV and alloantigens. Antigen-specific cells were identified using intracellular cytokine staining and HLA multimers. Peptide stimulation was used to induce cytokine expression, alongside control conditions (actin peptides and staphylococcal enterotoxin B). As these experiments focused on rare antigen specific populations, T cell specificity was determined by comparison of cytokine-expressing populations following experimental peptide library stimulation with the control conditions.
Gating strategy	Gating was established using our laboratory's established protocol and gating template. Full gating strategy is now added as Supplemental Figure 6.

- [x] Tick this box to confirm that a figure exemplifying the gating strategy is provided in the Supplementary Information.
